# Endothelial dysfunction in patients with granulomatosis with polyangiitis: a case–control study

**DOI:** 10.1007/s00296-018-4061-x

**Published:** 2018-05-30

**Authors:** Renata Pacholczak, Stanisława Bazan-Socha, Teresa Iwaniec, Lech Zaręba, Stan Kielczewski, Jerzy A. Walocha, Jacek Musiał, Jerzy Dropiński

**Affiliations:** 1 Department of Anatomy, Jagiellonian University, Medical College, Cracow, Poland; 2 Centre of Oncology, Maria Sklodowska-Curie Memorial Institute, Cracow Branch, Cracow, Poland; 3 2nd Department of Internal Medicine, Jagiellonian University, Medical College, ul.Skawińska 8, 31-066 Cracow, Poland; 4 Faculty of Mathematics and Natural Sciences, University of Rzeszow, Rzeszow, Poland

**Keywords:** Endothelium, Atherosclerosis, Systemic vasculitis, Ultrasonography

## Abstract

**Background:**

Granulomatosis with polyangiitis (GPA) is a rare granulomatous vasculitis affecting small- and medium-sized blood vessels. In optimally treated patients with long-standing disease, the common cause of death is atherosclerosis even in the absence of typical risk factors.

**Objective:**

To evaluate endothelial dysfunction in GPA patients.

**Methods:**

44 patients (21 men and 23 women) diagnosed with GPA and 53 controls matched for age, sex, BMI and typical risk factors for cardiovascular diseases (22 men and 31 women) were enrolled in the study. We measured each participant’s serum levels of vascular cell adhesion molecule-1 (VCAM-1), interleukin 6 (IL-6), and thrombomodulin. We also studied flow-mediated dilatation (FMD) of the brachial artery, intima-media thickness (IMT) of the common carotid artery and aortic stiffness using echocardiography.

**Results:**

Patients with GPA showed a 15.9% increase in serum levels of VCAM-1 (*p* = 0.01), 66% of IL-6 (*p* < 0.001) and 50.9% of thrombomodulin (*p* < 0.001) compared to controls. FMD% was 48.9% lower in patients with GPA in comparison to controls (*p* < 0.001), after adjustment for potential confounders, with no differences regarding IMT or aortic stiffness. FMD% was negatively associated with duration of the disease (*β* = − 0.18 [95% CI: − 0.32 to − 0.04]), C-reactive protein (*β* = − 0.17 [95% CI: − 0.27 to − 0.07]), IL-6 (*β* = − 0.29 [95% CI: − 0.39 to − 0.19]), blood creatinine level (*β* = − 0.2 [95% CI: − 0.3 to − 0.1]), and IMT (*β* = − 0.14 (− 0.24 to − 0.04). In a multiple linear regression model, kidney function, IMT, pack-years of smoking, diabetes and level of VCAM-1 were independent predictors of lower FMD%.

**Conclusion:**

GPA is characterized by endothelial dysfunction. FMD is a useful tool for the detection of endothelial injury.

**Electronic supplementary material:**

The online version of this article (10.1007/s00296-018-4061-x) contains supplementary material, which is available to authorized users.

## Introduction

Endothelium plays a key role in vascular homeostasis. It acts as a barrier between tissues and circulating blood and as a signal transducer that regulates vasomotor activity [1]. Activation of endothelial cells leads to upregulation of adhesion molecules, such as P-selectin, E-selectin, intercellular adhesion molecule-1, and vascular cell adhesion molecule-1 (VCAM-1), resulting in attachment and migration of circulating leukocytes. Differentiation of migrated monocytes into macrophages and the subsequent uptake of lipids by these cells results in foam cell generation and fatty streak formation. Further recruitment of inflammatory cells and proliferation of smooth muscle cells leads to the development of atherosclerotic plaque [2]. Endothelial function can be determined with a noninvasive ultrasound measurement of flow-mediated dilatation (FMD) of a brachial artery. This is the direct measurement of the arterial endothelium’s response to hyperemia (shear stress) that leads to the nitric oxide release and vasodilatation.

Granulomatosis with polyangiitis (GPA, formerly Wegener’s granulomatosis) is the common vasculitis with a prevalence of 3 per 100, 000 and peak incidence at the age of 50–60. Anti-neutrophil cytoplasmic antibodies (ANCA) are considered as the marker of the disease and are targeted against proteinase 3 (PR3). Its most common clinical features are granulomatous lesions of the upper and lower airways accompanied by the kidney failure [3, 4]. Cardiac involvement is infrequent in GPA, but coronary heart disease, arrhythmias, pericarditis, and nonbacterial thrombotic endocarditis can be present in these patients [5–8].

The increased morbidity from ischemic heart disease in GPA suggests that not only small vessels but also big ones are affected [9].

Atherosclerosis and its complications are one of the leading cause of death even in properly treated patients with long-standing ANCA-associated vasculitis (AAVs) [10, 11]. However, the mechanism by which atherosclerosis is promoted in these diseases is not explained by the classical atherosclerotic risk factors and remains under investigation.

The relationship between inflammation, vascular dysfunction and atherosclerosis is well-established. Premature atherosclerosis has been observed in patients with chronic inflammatory diseases such as systemic lupus erythematosus [12, 13], systemic sclerosis [14, 15] and antiphospholipid syndrome [16, 17]. There is currently a lack of reliable data on endothelial injury and development of premature atherosclerosis in the setting of vasculitis.

Previously, a few studies have analyzed the endothelial damage and progression of atherosclerosis in patients with systemic vasculitis [18–21]. Their results, however, were inconsistent and limited in significance due to the small number of subjects studied. For this reason, we sought to evaluate ultrasonographic and laboratory markers of endothelial injury in patients with GPA, which might be related to the premature and accelerated atherosclerosis and increased risk of cardiovascular events.

## Methods

A case–control observational, retrospective study was carried out with approval of the Bioethics Committee of XXXX University Medical College (9th May 2013, number of protocol: KBET/79/B/2013). The patients were recruited from the population of patients at the Department of Allergy and Clinical Immunology at the University Hospital in XXXXX in the period between 2014 and 2017 who were in the disease flare or symptomatic. The control group was enrolled from the hospital personnel and relatives. They were selected according to matching criteria. All participants received a detailed brief of the methodology and safety protocols for the study and provided written consent for their participation.

### Study groups

The case group constituted 44 patients with GPA—21 men and 23 women.

The control group consist of 53 individuals, 22 men and 31 women, matched to GPA patients by gender, age, body mass index (BMI) and smoking habit, as well as comorbidities, including hypertension, hypercholesterolemia, and diabetes mellitus.

### Patients

Each patient had a current or previous diagnosis of GPA based on the criteria of the American College of Rheumatology [22]. We analyze only patients with the disease flare or those, who were symptomatic and diagnosed with persistent disease. Disease activity was measured using the Birmingham Vasculitis Activity Score (BVAS) [23]. Disease flare was defined as the presence of new symptoms (major or minor item in BVAS). Persistent disease was defined as the presence of one or more persistent symptoms attributed to active disease for more than 1 month but less than 3 months. For symptoms which occurred in patients since the onset of GPA and were present for more than 3 months, we used Vascular Damage Index (VDI) [24]. Patients with congestive heart failure, coronary heart disease, uncontrolled hypertension, liver failure, and cancer were excluded from the study (for details see supplementary material).

### Main outcome variable

In this case–control study, we analyzed whether GPA is associated with vascular endothelial damage. We measured flow-mediated dilatation of the brachial artery, intima-media thickness of the common carotid artery, aortic stiffness as well as evaluated serum levels of thrombomodulin and VCAM-1 in GPA patients and matched control subjects.

### Procedures

#### Laboratory analysis

Fasting blood samples were drawn in the morning from the antecubital vein using minimal stasis. Lipid profile, glucose, liver enzymes, urine, creatinine with eGFR, complete blood cell and platelet count were analyzed by routine laboratory techniques. C-reactive protein (CRP) was measured using analyzer VITROS 250 Johnson & Johnson. Blood samples were drawn into serum separation tubes, centrifuged at 2000×g for 20 min at room temperature, within 2 h from sampling. The supernatant was frozen in aliquots and stored at -70 °C until analysis. Interleukin-6 (IL-6), VCAM-1, and soluble thrombomodulin were measured using standardized ELISA method (all, R&D Systems, Minneapolis, MN, USA). Anti-PR3 IgG was measured in all GPA subjects using ELISA assay (EUROIMMUN, Lübeck, Germany).

#### Ultrasound examinations

Ultrasound examinations were performed in a darkened, quiet, room, after at least 10 min rest in a supine position, using high-quality ultrasonograph (Sequoia 512 with a 10 MHz linear array ultrasonic transducer, MountainView, Ca, USA). Before examination, the subjects refrained from eating for at least 10 h. Examinations were conducted by two independent ultrasonography experts and considered parameters constituted a mean of three subsequent measurements. A complete transthoracic echocardiogram (TTE) was performed in every participant with estimation of ejection fraction of the left ventricle (EF) and systolic pulmonary artery pressure in accordance to standard methods [25]. Flow-mediated dilatation (FMD) of the brachial artery was measured in accordance to Celermayer’ method [26]. Aortic stiffness was expressed as a percentage of aortic systolic diameter (ASD) and aortic diastolic diameter (ADD): aortic stiffness % = [(ADD − ASD)/ASD] × 100%. The intima-media thickness (IMT) of the carotid artery was also measured and in the further analysis we used a mean value of the IMT measured on the right and left common carotid artery (for details see supplementary material).

### Statistical analysis

The results were compared between the case and control groups using STATISTICA 12.5 Software. Continuous variables, all non-normal distributed values (verified by the Shapiro–Wilk test), were given as median and interquartile range and compared by the Mann–Whitney *U* test. Categorical variables were presented as numbers (percentages) and compared by *χ*^2^ test. Potential confounders were identified as: age, BMI, sex, and comorbidities such as arterial hypertension, diabetes mellitus and hypercholesterolemia. To adjust for these, obtained results of FMD%, IMT, aortic stiffness%, IL-6, VCAM-1, and thrombomodulin were log-transformed and a one way covariance analysis (ANCOVA) was performed, to achieve the overall *p* value. The univariate linear regression tests (with adjustment for aforementioned confounders) were used to analyze associations between two selected parameters. Independent determinants of FMD% were established in multiple linear regression model, built by a forward stepwise selection procedure, verified by F Snedecore’s statistics, with *F* > 1. The *R*^2^ was used as a measure of the variance. To calculate odds ratios (ORs) with 95% confidence intervals (CIs), unconditional multivariate logistic regression was performed. The cut-off values for IL-6, VCAM-1, FMD%, and thrombomodulin were determined based on receiver operating characteristic (ROC) curves. Results were considered statistically significant when the *p* value was less than 0.05.

## Results

### Characteristics of patients and controls

Demographic, clinical and laboratory characteristics of the studied subjects, including basic laboratory tests, ultrasound parameters, and cardiovascular risk factors were given in Table 1. Both groups were similar in age, sex, BMI as well as prevalence of comorbidities (hypercholesterolemia, hypertension, and diabetes mellitus), smoking habit, and family history of cardiovascular diseases. Parameters describing GPA activity, as well as current and past therapy were given in Table 2. The median duration of the disease was 4.5 years. More than half of the patients had active disease at the time of evaluation. All of them had detectable anti-PR3. Most of them were being treated with steroids currently or in the past with other immunosuppressive agents, such as: azathioprine, cyclophosphamide, methotrexate, mycophenolate mofetil and rituximab. Additionally, GPA patients were receiving statins, beta-blockers, angiotensin-converting enzyme inhibitors or angiotensin receptor antagonists, diuretics and calcium channel blockers. Lungs were the most commonly involved organs, followed by paranasal sinuses and kidneys.

**Table 1:** A summary of demographic, laboratory and echocardiographic parameters in patients with granulomatosis with polyangiitis and controls

	Patients, n* = 44	Controls, *n* = 53	*p* value
Age (years)	59 (46–65)	48 (43–61)	0.07
Male gender, number (%)	21 (47.6)	22 (41.5)	0.67
Body mass index (kg/m2)	26.1 (24.1–29.6)	26.6 (23.9–29.1)	0.93
Basic laboratory tests			
Hemoglobin (g/dl)	12.25 (10.55–13.55)	13.7 (12.7–15)	< 0.001^a^
Red blood cells (10^3^/ul)	4.13 (3.7–4.5)	4.5 (4.2–4.9)	< 0.001^a^
White blood cells (10^3^/ul)	7.46 (5.76–10.06)	5.9 (5.03–6.96)	< 0.001^a^
Platelet count (10^3^/ul)	235.5 (171–287)	225 (200–275)	0.67
Total cholesterol (mmol/l)	4.7 (3.9–5.4)	4.9 (4.2–5.25)	0.58
Low-density lipoprotein (mmol/l)	2.4 (1.9–3.2)	3.1 (2.5–3.6)	0.003^a^
Triglycerides (mmol/l)	1.7 (1.2–2.1)	1.1 (0.7–1.5)	0.002^a^
Glucose (mmol/l)	5 (4.45–5.43)	4.95 (4.72–5.2)	0.95
Creatinine (mmol/l)	101.9 (72.5–240)	76.1 (68.3–90)	0.01^a^
Urea (mmol/l)	7.55 (5.8–12.7)	4.56 (3.93–5.3)	< 0.001^a^
Estimated glomerular filtration rate (ml/min/1.73 m^2^)	60 (26–67)	60 (60–80)	0.01^a^
Alanine transaminase (U/l)	21.5 (15–32)	22.5 (14–28)	0.72
C-reactive protein (mg/dl)	7.6 (5–19.4)	1.2 (1–2.1)	< 0.001^a^
Interleukin-6 (pg/ml)	5.03 (3.02–10.5)	1.7 (1.08–2.16)	< 0.001^a^
Echocardiographic parameters			
Left ventricular diastolic diameter (cm)	4.8 (4.6–5.3)	4.7 (4.5–4.9)	0.29
Left ventricular systolic diameter (cm)	3 (3–3.4)	3 (2.9–3.1)	0.049^a^
Right ventricular diameter (cm)	2.2 (2–2.3)	2.1 (1.9–2.3)	0.01^a^
Left atrial diameter (cm)	3.9 (3.7–4.1)	3.7 (3.5–3.9)	0.004^a^
Left ventricle posterior wall thickness (cm)	1.05 (0.9–1.2)	0.9 (0.8–1)	< 0.001^a^
Interventricular septum thickness (cm)	1.1 (1–1.2)	0.9 (0.8–1)	< 0.001^a^
Ejection fraction (%)	65 (60–68)	68 (68–70)	< 0.001^a^
Pulmonary artery pressure (mmHg)	32 (30–36)	32 (26–32)	0.01^a^
Laboratory parameters of endothelial injury			
Vascular cell adhesion molecule-1 (ng/ml)	957 (749.1–1273.4)	804.6 (694.4–936.7)	0.01^a^
Thrombomodulin (ng/ml)	8.9 (5.2–1.4)	4.3 (3.9–4.7)	< 0.001^a^
Ultrasound parameters of endothelial injury and atherosclerosis			
Relative increase of flow-mediated dilatation of a brachial artery	5.26 (4.08–8.01)	10.3 (8.89–12,5)	< 0.001^a^
Aortic stiffness (%)	7.14 (4–9.09)	7.4 (6.25–10.34)	0.27
Median value of intima-media thickness of a common carotid artery (cm)	0.07 (0.06–0.08)	0.07 (0.06–0.08)	0.20
Other cardiovascular risk factors			
Hypertension n(%)	21 (50)	16 (30.2)	0.08
Diabetes mellitus n(%)	9 (21.43)	6 (11.3)	0.2
Hypercholesterolemia n(%)	14 (33.33)	19 (35.8)	0.68
Smoking currently n(%)	3 (7.14)	4 (7.55)	0.9
In the past *n*(%)	13 (30.95)	15 (28.3)	0.33
Smoking (packs/years)	0 (0–15)	0 (0–3)	0.64
Positive family history of cardiovascular diseases n(%)	9 (21.42)	7 (13.2)	0.32

^a^Categorical variables are presented as numbers (percentage), continuous variables as median and interquartile range. The results which are statistically significant are marked

**Table 2:** Clinical characteristics of the patients (*n* = 44) with granulomatosis with polyangiitis

	Patients
Duration of the disease (years)	4.5 (1–9)
Active disease *n* (%)	26 (59.1)
BVAS in active disease	9 (8–10)
Persistent disease n(%)	16 (36.36)
BVAS in persistent disease	4 (3–5)
Anti-proteinase 3 antibodies (IU/ml)	20.5 (5–65)
VDI score in eligible patients	3 (0–5)
Organ involvement	
Cutaneous vasculitis *n* (%)	13 (30.95)
Granulomatous lesions in ears/hearing disturbances *n* (%)	11 (26.19)
Granulomatous lesions in larynx *n* (%)	6 (14.63)
Paranasal sinuses inflammation *n* (%)	30 (71.42)
Bone destruction of paranasal sinuses n(%)	16 (38.1)
Chronic kidney disease *n* (%)	22 (52.38)
Lungs *n* (%)	31 (73.81)
Peripheral nerves *n* (%)	10 (23.8)
Gastrointestinal system *n* (%)	1 (2.38)
Heart *n* (%)	1 (2.38)
Treatment characteristic	
Current steroids *n* (%)	37 (88.1)
Current steroids dose (mg/day of prednisone)	8 (4–20)
Systemic steroids therapy (years)	2 (0.5–5)
Immunosuppressive treatment (currently or in the past)	
Azathioprine *n* (%)	12 (28.57)
Cyclophosphamide *n* (%)	37 (88.1)
Total dose of cyclophosphamide (grams)	8.15 (3.9–19)
Methotrexate *n* (%)	5 (11.9)
Mycophenolate mofetil *n* (%)	2 (5.26)
Rituximab *n* (%)	13 (30.95)
Internal medicine medications	
Angiotensin-converting enzyme inhibitors or angiotensin receptor antagonists *n* (%)	12 (28.57)
Statins *n* (%)	21 (51.22)
Beta-blockers *n* (%)	17 (40.48)
Diuretics *n* (%)	12 (28.57)
Calcium channel blockers *n* (%)	12 (28.57)

Categorical variables are presented as numbers (percentage), continuous variables as median and interquartile range

*n* number, *BVAS* Birmingham Vasculitis Activity Score, *VDI* vascular damage index

### Basic laboratory tests and basic transthoracic echocardiographic parameters

As expected, GPA patients were characterized by higher inflammatory markers, such as CRP, IL-6 (reference range: 0.45–9.96 pg/ml) and white blood cells, as well as impaired kidney function and lower hemoglobin level (Table 1). Moreover, there were characterized by higher triglycerides.

In TTE GPA subjects had larger left and right ventricles and left atria, thicker posterior walls and interventricular septa, as well as lower ejection fraction and higher systolic pulmonary artery pressure.

### Laboratory markers of endothelial injury

GPA patients had a 15.9% higher levels of VCAM-1 (*p* = 0.01) and a 50.9% increased thrombomodulin concentrations (*p* < 0.001) in peripheral blood, comparing to healthy individuals. However, in ANCOVA analysis we documented that only thrombomodulin levels remained higher in GPA subjects after adjustment for potential confounders (age, sex, BMI, hypercholesterolemia, hypertension, and diabetes mellitus, *p* < 0.001). The VCAM-1 was similar in GPA and control groups in this analysis (*p* = 0.54). Moreover, patients with GPA had increased risk of elevated VCAM-1 (OR 5.75 [95% CI: 2–16.38], reference range: 349–991 ng/ml), and thrombomodulin (OR 6.71 [95% CI: 3.37–13.3], reference range: 2.9–5.3 ng/ml) compared to the healthy individuals (cut-off points: 1213.96 and 5.9 ng/ml, respectively). As expected, both endothelial injury markers were related to the CRP (*β* = 0.18 [95% CI: 0.08–0.28], and *β* = 0.28 [95% CI: 0.27–0.29], VCAM-1 and thrombomodulin, respectively) and IL-6 level (*β* = 0.27 [95% CI: 0.15–0.39], and *β* = 0.4 [95% CI: 0.27–0.53], VCAM-1 and thrombomodulin, respectively). Moreover, we demonstrated strong positive association between white blood cells and thrombomodulin (*β* = 0.2 [95% CI: 0.11–0.29]). Table 3 demonstrates the most important associations of selected laboratory and echocardiographic parameters after adjustment for confounders with linear regression models. We documented positive associations between laboratory parameters of endothelial damage and kidney function, anti-PR3 level as well as interventricular septum and posterior wall thickness (see Table 3).

**Table 3:** Correlations of selected laboratory and echocardiographic parameters in GPA patients

	Flow-mediated dilatation% *β* (95% CI)	Intima-media thickness (cm) *β* (95% CI)	Aortic stiffness%, *β* (95% CI)	Vascular cell adhesion molecule-1 (ng/ml), *β* (95% CI)	Trombomodulin (ng/ml), *β* (95% CI)
White blood cells (10^3^/ul)	− 0.24 (− 0.32 to − 0.15)*	0.07 (− 0.02 to 0.16)	− 0.23 (− 0.36 to − 0.1)*	0.08 (− 0.02 to 0.18)	0.2 (0.11 to 0.29)*
C-reactive protein (mg/dl)	− 0.17 (− 0.27 to − 0.07)*	− 0.03 (− 0.13 to 0.07)	− 0.19 (− 0.31 to − 0.7)*	0.18 (0.08 to 0.28)*	0.28 (0.27 to 0.29)*
Interleukin-6 (pg/ml)	− 0.29 (− 0.39 to − 0.19)*	− 0.03 (− 0.13 to 0.07)	0.08 (− 0.06 to 0.22)	0.24 (0.14 to 0.34)*	0.29 (0.2 to 0.38)*
Creatinine(mmol/l)	− 0.2 (− 0.3 to − 0.1)*	0.18 (0.08 to 0.28)*	0.27 (0.15 to 0.39)*	0.39 (0.29 to 0.49)*	0.6 (0.53 to 0.67)*
Urea(mmol/l)	− 0.06 (− 0.16 to 0.04)	0.11 (0 to 0.22)	0.11 (− 0.04 to 0.26)	0.27 (0.06 to 0.38)*	0.63 (0.55 to 0.71)*
Estimated glomerular filtration rate (ml/min/1.73 m^2^)	0.05 (− 0.05 to 0.15)	− 0.12 (− 0.24 to 0)	0.00 (− 0.19 to 0.19)	− 0.23 (− 0.36 to − 0.1)*	− 0.48 (− 0.38 to − 0.58)*
Interventricular septum thickness (cm)	− 0.23 (− 0.33 to − 0.13)*	0.12 (0.01 to 0.23)*	0.01 (− 0.13 to 0.15)	0.29 (0.17 to 0.41)*	0.43 (0.33 to 0.53)*
Posterior wall thickness (cm)	− 0.29 (− 0.39 to − 0.19)*	0.16 (0.05 to 0.27)*	0.00 (− 0.14 to 0.14)	0.35 (0.24 to 0.46)*	0.5 (0.41 to 0.59)*
Flow-mediated dilatation%	–	− 0.12 (− 0.22 to − 0.02)*	0.25 (0.1 to 0.4)*	0.03 (− 0.09 to 0.15)	− 0.07 (− 0.17 to 0.03)
Intima-media thickness (cm)	− 0.14 (− 0.24 to − 0.04)*	–	0.00 (− 0.18 to 0.18)	− 0.09 (− 0.22 to 0.04)	0.05 (− 0.05 to 0.15)
Aortic stiffness%	0.2 (− 0.07 to 0.43)	0.00 (− 0.12 to 0.12)	–	0.31 (0.17 to 0.44)*	0.16 (0.03 to 0.29)*
Vascular cell adhesion molecule-1 (ng/ml)	0.02 (− 0.08 to 0.12)	− 0.08 (− 0.18 to 0.02)	0.3 (0.17 to 0.43)*	–	0.57 (0.49 to 0.65)*
Thrombomodulin (ng/ml)	− 0.09 (− 0.19 to 0.01)	0.05 (− 0.05 to 0.15)	0.19 (0.04 to 0.34)*	0.72 (0.62 to 0.82)*	–
Smoking (packs/years)	− 0.33 (− 0.44 to − 0.12)*	0.04 (− 0.02 to 0.1)	− 0.17 (− 0.47 to 0.13)	− 0.2 (− 0.47 to 0.07)	− 0.04 (− 0.3 to 0.2)
Steroids time of treatment (years)	− 0.07 (− 0.23 to 0.1)	0.19 (0.03 to 0.35)*	− 0.19 − 0.43 to 0.05	− 0.1 − 0.29 to 0.09	0.05 − 0.13 to 0.22
Duration of the disease	− 0.18 (− 0.32 to − 0.04)*	0.27 (0.14 to 0.41)*	0.13 (− 0.08 to 0.34)	− 0.1 (− 0.28 to 0.08)	0.09 (− 0.06 to 0.24)
Concentration of anti-proteinase 3 antibodies (IU/ml)	0.00 (− 0.14 to 0.14)	− 0.08 (− 0.23 to 0.07)	− 0.04 (− 0.24 to 0.16)	0.19 (0. 02 to 0.36)*	0.163 (0.001 to 0.33)*

The resulting regression coefficients (*β*) were given after adjustment for age, sex, BMI, and comorbidities (hypertension, diabetes mellitus and hypercholesterolemia)

*The results which are statistically significant are marked

GPA patients with hypertension had 30. 6% higher levels of VCAM-1 (1178.3 [815.2–1600.1] vs. 893.8 [635.4–959.8] ng/ml, *p* = 0.02) and 41% higher thrombomodulin levels (11.3 [7.8–17.6] vs. 5.9 [4.4–9.5] ng/ml, *p* = 0.01) than the remaining GPA patients. Moreover, GPA patients with chronic kidney disease were characterized by higher thrombomodulin level (11.7 [9.4–17.6) vs. 5.4 [4.4–6.9] ng/ml, *p* < 0.001) and VCAM-1 level (1258.2 [893.2–1457.7] vs. 747.4 [546.9–917.5] ng/ml, *p* < 0.001). Other comorbidities had no impact on laboratory markers of endothelial damage. Patients taking statins and antihypertensive medications had increased thrombomodulin blood level (10.7 [7.5–15.2] vs. 5.6 [4.1–11.9] ng/ml, *p* = 0.03, and 11.1 [7.1–16.1] vs. 5.4 [3.9–8.1] ng/ml, *p* = 0.01, respectively), Moreover, those treated with antihypertensives had higher level of VCAM-1 (1089.7 [884.5–1293.1] vs. 803.4 [520.1–905.3] ng/ml, *p* = 0.01).

In the subgroups analysis, patients in persistent disease or in active disease had similar results of laboratory parameters of endothelial injury (thrombomodulin 12.63 [5.99–18.86] vs. 10.18 [5.24–11.4] ng/ml, *p* = 0.19, VCAM-1 1099.05 [863.94–1263.64] vs. 1065.82 [745.64–1273.38] ng/ml, *p* = 0.58).

### Ultrasound parameters of endothelial injury

GPA patients had 48.9% decrease in FMD% compared to controls (*p* < 0.001, also after adjustment for potential confounders: age, sex, BMI, hypercholesterolemia, hypertension, diabetes mellitus *p* < 0.001), and markedly higher risk of diminished FMD% defined as values below the cut-off point of 8.51 (OR 4.9 [95% CI: 2.88–8.23]).

In Table 3 are given selected associations of FMD% with other laboratory and ultrasound parameters. As presented, FMD% was negatively associated with white blood cells (*β* = − 0.24 [95% CI: − 0.32 to − 0.15]), CRP (β = − 0.17 [95% CI: − 0.27 to − 0.07]), IL-6 (*β* = − 0.29 [95% CI: − 0.39 to − 0.19]) and the blood creatinine level (*β* = − 0.2 [95% CI:− 0.3 to − 0.1]) in univariate linear regression models. Interestingly, FMD% was also negatively related to smoking (packs/years) (*β* = − 0.33 [95% CI: − 0.44 to − 0.12)], duration of the disease (*β* = − 0.18 [95% CI: − 0.32 to − 0.04]), as well as posterior wall and interventricular septum thickness (*β* = − 0.29 [95% CI: − 0.39 to − 0.19], *β* = − 0.23 [95% CI: − 0.33 to − 0.13], respectively).

A multiple regression model showed that various factors independently determined FMD%, including presence of diabetes mellitus (*β* = − 0.41 [95% CI: − 0.55 to − 0.27]), pack-years of smoking (*β* = − 0.14 [95% CI: − 0.29 to − 0.01]), IMT (*β* = − 0.34 [95% CI: − 0.5 to − 0.18]), serum urea (*β* = − 0.41 [95% CI: − 0.61 to − 0.21]) or VCAM-1 (*β* = − 0.33 [95% CI: − 0.53 to − 0.13]), (Table 4).

**Table 4:** Multiple linear regression model for a relative increase of flow-mediated dilatation of a brachial artery comparing patients and control group

	Patients		Control	
	*β* (95% CI)	*R* ^2^	*β* (95% CI)	*R* ^2^
Duration of the disease (years)	0.15 (− 0.08 to 0.38)	0.51	–	0.32
Total cholesterol level (mmol/l)	− 0.06 (− 0.19 to 0.07)		0.07 (− 0.05 to 0.19)	
Urea (mmol/l)	− 0.41 (− 0.61 to − 0.21)*		− 0.22 (− 0.36 to − 0.08)*	
Posterior wall thickness (cm)	0.03 (− 0.15 to 0.21)		− 0.41 (− 0.56 to − 0.26) *	
Intima-media thickness of a common carotid artery (cm)	− 0.34 (− 0.50 to − 0.18)*		− 0.20 (− 0.35 to − 0.05)*	
Total dose of cyclophosphamide (grams)	− 0.19 (− 0.41 to 0.03)		–	
Smoking (packs/years)	− 0.14 (− 0.29 to − 0.01)*		–	
Diabetes mellitus	− 0.41 (− 0.55 to − 0.27)*		–	
Vascular cell adhesion molecule-1 (ng/ml)	− 0.33 (− 0.53 to − 0.13)*		− 0.09 (− 0.22 to 0.04)	
Adjustment statistics	*F* = 2.84, *p* = 0.01		*F* = 4.2, *p* < 0.001	

The resulting standardized regression coefficient (*β*) with 95% confidence intervals (95% CI) for a factor (independent variable) indicates the increase/decrease in standard deviations (SDs) of dependent variable, when that particular factor increases with 1 SD and all other variables in the model are unchanged. The results which are statistically significant are marked *

Among GPA patients, lower FMD% was observed in those with hypertension (5 [2.9–5.9] vs. 6.4 [4.7–8.9], *p* = 0.01), diabetes (3.4 [2.9–5] vs. 6 [4.3–8.4], *p* = 0.01) and smoking currently or in the past (4.3 [2.9–5] vs. 6.7 [5–0.3]). Interestingly, only those treated with azathioprine had lower FMD% (4.13 [2.9–5.13] vs. 6.7 [4.9–8.4], *p* = 0.01) without influence of other immunosuppressive drugs. Patients treated with statins were also characterized by decrease in FMD% (4.5 [2.9–5.3] vs. 6.8 [5.1–9.4], *p* = 0.01).

The values of aortic stiffness% and IMT were similar in both studied groups. Aortic stiffness% and IMT did not correlate with laboratory parameters of endothelial injury. However, IMT was related to FMD% (*β* = − 0.12 [95% CI: − 0.22 to − 0.02]), blood creatinine level (*β* = 0.18 [95% CI: 0.08 to 0.28]), duration of steroid treatment (*β* = 0.19 [95% CI: 0.03 to 0.35]) duration of the disease (*β* = 0.27 [95% CI: 0.14 to 0.41]), posterior wall thickness (*β* = 0.16 [95% CI:0.05 to 0.27]), and interventricular septum thickness (*β* = 0.12 [95% CI:0.01 to 0.23]) (Table 3).

In the subgroup of GPA patients with chronic kidney disease IMT was higher (0.08 [0.06–0.09] vs. 0.065 [0.055–0.075], cm *p* = 0.01). GPA subjects treated with statins and with hypertension had higher IMT than remaining GPA patients (0.08 [0.07–0.09] vs. 0.06 [0.05–0.07] cm, *p* = 0.01, and 0.08 [0.07–0.09] vs. 0.06 [0.06–0.07] cm, *p* = 0.001, respectively). Only one patient had an atherosclerotic plaque in the left common carotid artery.

Aortic stiffness% was negatively associated with blood leukocyte count (*β* = − 0.23 [95% CI: − 0.36 to − 0.1] and CRP level (*β* = − 0.19 [95% CI: − 0.31 to − 0.7]. Comorbidities and medication had no impact on aortic stiffness%.

Ultrasound parameters of endothelial injury were comparable between the patients in the disease flare and in the persistent disease (FMD% 5.92 [3.35–8.89] vs. 5.86 [4.35–7.14], *p* = 0.76, IMT 0.07 [0.06–0.09] vs. 0.07 [0.06–0.08] cm, *p* = 0.92, aortic stiffness% 7.93 [4.56–10.53] vs. 7.69 [3.85–8.82], *p* = 0.4).

## Discussion

This study demonstrates that GPA patients suffer vascular endothelial damage that is manifested by increased serum levels of thrombomodulin and VCAM-1, as well as lower flow-mediated dilatation of the brachial artery. Both the laboratory markers of endothelial injury increase while FMD decreases in association with inflammatory markers, such as IL-6 and CRP. These observations suggest that the most important predictor of endothelial damage in GPA is a persistent systemic inflammatory state. However, multiple regression analysis shows that impaired FMD was also independently determined by other factors, including kidney insufficiency, diabetes as well as smoking habit.

The associations of laboratory and ultrasound parameters of endothelial damage with markers of inflammation may indicate that the process of endothelial injury is more prominent in active phase of the disease. However, comparing the subgroups in persistent disease or in active disease these parameters did not differ regardless of phase of the disease. According to Tervaert [27] atherosclerosis is accelerated in the active phase but endothelial function returns to normal when inflammation is pharmacologically controlled. Another study showed that successful immunosuppressive treatment improves endothelial function, reaffirming the key role of inflammation in pathogenesis of atherosclerosis in this disease [28]. Such thesis was also recognized in children with primary systemic vasculitis without common cardiovascular risk factors [29]. Moreover, Nienhuis et al. [21] demonstrated impaired endothelium-dependent vasodilatation in microcirculation of the hand vessels of GPA patients, even if they did not have clinical manifestations of atherosclerosis.

In our study, we did not document any significant differences in IMT between GPA patients and healthy controls. This observation stays in line with results published by De Souza et al. [30], who suggested that premature atherosclerosis in GPA patients might be postponed by use of statins or prednisolone. This contradicts other reports [21, 31] that have shown that GPA patients are characterized by higher IMT, indicating accelerated atherosclerosis. Potential discrepancy between these results may be due to the small sample size, different treatment mode or duration of the disease in evaluated patients. Importantly in our study, we found that IMT related positively to the time course of GPA.

The reason of endothelial dysfunction in GPA remains unknown. However, it seems that it might be related to the pathogenesis of the disease and eventually its therapeutic possibilities. In our study, the level of anti-PR3 antibodies was related to markers of endothelial injury. As it has been shown, autoantibodies in AAVs activate neutrophils which then adhere to the inner vessel wall causing endothelial impairment by release of proteolytic enzymes and triggering vasculopathic cascade [32, 33]. Moreover, pro-inflammatory cytokines increased in active systemic vasculitis depress endothelium-dependent relaxation in vitro, as well as in vivo [34], while reactive oxygen species lead to oxidation of lipoproteins which are responsible for endothelial cell injury [35].

In the study by Clarke et al. [29] the level of endothelial damage biomarkers was predominantly affected by disease activity rather than by treatment. However, it has been shown that cyclophosphamide, a cytotoxic drug used to induce remission in AAVs patients, directly injures endothelial cells leading to subsequent leakage of plasma to the extravascular space [36]. Colleoni et al. [37] observed a significant drop in the level of vascular endothelial growth factor in breast cancer patients after oral administration of cyclophosphamide in small doses, which also suggests its anti-angiogenic effect. This finding was also confirmed by Folkman et al. [38], who found that systemic administration of cyclophosphamide, anthracyclines or paclitaxel, inhibits neovascularization in the mouse cornea. In cancer treatment, cyclophosphamide is used in higher doses than in AAVs, however, it should be considered as an additional factor of potential endothelial damage. In our study, we did not observe differences in parameters of endothelial injury in those patients treated vs. those not treated with cyclophosphamide or other immunosuppressive drugs, such as methotrexate, mycophenolate mofetil, and rituximab (data not shown). Only in those treated with azathioprine the FMD% value was lower. There are just a few reports describing potential role of azathioprine in endothelial cell injury [39, 40]. However, the majority of the GPA patients were also treated with steroids. Prolonged steroid therapy is associated with hypertension, diabetes mellitus and change in the lipid profile, all of which influence the risk of atherosclerosis and cardiovascular events [41].

It has been demonstrated that renal insufficiency might be related to the endothelial dysfunction in other autoimmune diseases [2] as well as in peritoneal dialysis [42]. Hypertension, one of the complications of kidney failure, is known to be implicated in increased arterial stiffness and endothelial dysfunction. Additionally, uremia has been considered as a nontraditional cardiovascular risk factor [43]. However, this relationship in GPA patients has not been described in previous reports [44]. In our study, we observed positive association of blood creatinine, as well as urea level with FMD%, VCAM-1, and thrombomodulin. GPA patients with chronic kidney disease also had increased levels of VCAM-1, thrombomodulin and IMT. This is a novel finding of our study. Urea level in GPA patients was also independent predictors of lower FMD% in a multiple linear regression model.

The lower FMD in subjects with GPA documented in our study is possibly related not only to the endothelial dysfunction, but also to the arteries wall remodeling. In the previous study the blood vessels of these patients showed an increased level of matrix metalloproteinases (markers of vascular remodeling) [31]. Moreover, we have found a negative correlation between FMD and interventricular septum and posterior wall thickness. We also demonstrated that patients with GPA are characterized by larger left and right ventricular diameters and left atrial diameter, as well as increased left ventricle posterior wall thickness and interventricular septum thickness. In our opinion, these findings are most likely related to the inflammatory process of the kidneys and lungs, the organs, most often affected by GPA. Kidney damage results in overload of the circulatory system, whereas the inflammatory process in the lungs leads to the pulmonary hypertension. Both of these processes lead to the secondary hypertrophy of the heart cavities, which we have found in our patients. Observed differences might be also related to the cardiovascular system involvement in course of GPA and vessel wall remodeling, leading to the increased stiffness of the arteries and higher afterload, as well as heart hypertrophy. These findings, however, require further investigation.

Smoking currently or in the past also influenced lower FMD in GPA patients and it was an independent predictor of lower FMD.

Described changes may lead to the increased risk of cardiovascular events, what has been previously demonstrated by Faurschou et al. [9]. In his study, patients with GPA had 1.9 (95% CI: 1.4–2.4) higher risk of cardiovascular disease. Based on this result, we may speculate that early detection of endothelial dysfunction in GPA patients may help in selecting the most suitable preventive strategy. Tervaert [27] suggested that patients with large-vessels vasculitis should be treated with aspirin (75–125 mg/day) to prevent ischemic complications. Statins should also be advised in most patients with GPA for endothelial protection. Obviously, patients benefit from optimal immunosuppressive treatment that controls inflammation and prevents from consequences of GPA. However, it is necessary to keep in mind that immunosuppressive treatment directly leads to endothelial cell injury.

## Study limitation

The limited number of GPA patients decreases the power of our findings. However, our study group is one of the biggest evaluated in the literature so far in terms of endothelial dysfunction. Moreover, the GPA is a rare disease and in our opinion every report is valuable. Patients with GPA had some comorbidities (diabetes mellitus, hypertension or kidney insufficiency), which in the majority of analyzed subjects were related to the systemic complications of vasculitis and might be considered as a consequence of GPA. We attempted to eliminate these confounding variables by an adjustment for comorbidities (hypercholesterolemia, hypertension, and diabetes mellitus) during statistical analysis and recruitment of controls with similar common cardiovascular risk factors. GPA patients were younger than controls, but this difference did not reach statistical significance. Finally, patients with vasculitis were being treated with many medications, notably immunosuppressive drugs and corticosteroids. The impact of the medications used on endothelial dysfunction was beyond the scope of our study; however, decreased values of FMD% in patients on statins and antihypertensive medications seemed to be related more to comorbidities (hypercholesterolemia, hypertension) than the drugs themselves. Nevertheless, we believe that the presented results reflect true intergroup differences.

## Conclusion

In summary, the patients with GPA are characterized by endothelial dysfunction, which is likely related to the chronic systemic inflammation observed in autoimmune diseases. Although large observational studies are needed to verify whether lower FMD% is associated with increased risk of cardiovascular events in GPA patients, this noninvasive and simple ultrasound test seems to represent a new tool/predictor of endothelial injury for clinical practice.

## Electronic supplementary material

Below is the link to the electronic supplementary material.

Supplementary material 1 (DOCX 13 KB)
