# Supplementary material for: Endothelial dysfunction in patients with granulomatosis with polyangiitis: a case–control study
Source: Rheumatol Int. 2018 May 30;38(8):1521–30. doi: 10.1007/s00296-018-4061-x (PMC6060787; doi:10.1007/s00296-018-4061-x)
Supplement: Supplementary file 1 — Supplementary material 1 (DOCX 13 KB) [file 296_2018_4061_MOESM1_ESM.docx]

**Supplementary material**

**Exclusion criteria**

Exclusion criteria were: congestive heart failure, coronary heart disease, uncontrolled hypertension, liver failure, and cancer during treatment. Arterial hypertension was defined as a history of hypertension (blood pressure > 140/90 mmHg) or current antihypertensive treatment and it was considered as uncontrolled when it was resistant to treatment. Congestive heart failure was defined as left ventricular ejection fraction below 40%. Coronary heart disease was diagnosed on a basis of symptoms of chest pain or myocardial infarct in the past. Liver injury was defined as serum alanine aminotransferase elevated at least twice above upper limit of the reference range.

**Detailed methodology of performed examinations**

**Brachial artery ultrasonography**

Flow-mediated dilatation (FMD) of the brachial artery was measured in accordance to Celermayer' method . A baseline sagittal diameter (D1) of a distal part of the brachial artery was measured in M-presentation by using a 10 MHz linear array ultrasonic transducer, around approximately 2-3 cm proximal to its bifurcation. Afterwards, a sphygmomanometer cuff was placed on the forearm below the elbow and inflated to a pressure of 200mmHg for 5 minutes. The brachial artery diameter was measured again (D2) at the same point as previously 1 minute after release of the cuff. FMD was defined as the increase of brachial artery diameter after deflation of the cuff and was expressed as a percentage of the average baseline diameter (FMD %= [(D2-D1)/D1] x 100%).

**Aortic stiffness**

Measurements of an aortic diameter were performed using a 4 MHz echocardiographic transducer. During this measurement, a full cardiac cycle was additionally recorded by electrocardiography (ECG). The aortic diameter was measured in the parasternal long axis in M-mode approximately 3 centimeters above the aortic valve. Aortic systolic diameter (ASD) was measured during a full opening of aortic valve and aortic diastolic diameter (ADD) at the peak of the QRS complex. Aortic stiffness was expressed as a percentage of ASD and ADD (Aortic stiffness %= [(ADD-ASD)/ASD] x 100%) .

**Intima-media thickness of the common carotid artery.**

The intima-media thickness (IMT) of the carotid artery was measured with a 10 MHz linear transducer by two-dimensional echocardiogram. The anterior and posterior walls of the common carotid artery in the longitudinal projection were measured in the right and left common carotid arteries immediately distal to their bifurcation. In the further analysis we used a mean value of the IMT measured on a right and left common carotid artery.
